# Editorial: IUIS junior community: pushing the frontiers of immunology

**DOI:** 10.3389/fimmu.2025.1768685

**Published:** 2026-01-13

**Authors:** Adeleye O. Adeshakin, Anne M. Hahn

**Affiliations:** 1 Department of Bone Marrow Transplantation and Cellular Therapy, St Jude Children’s Research Hospital, Memphis, TN, United States; 2 Department of Microbiology and Immunology, University of Melbourne, at the Peter Doherty Institute for Infection and Immunity, Melbourne, VIC, Australia

**Keywords:** bioinformatics, cellular therapy, epidemiology, innate & adaptive immune response, vaccination

The International Union of Immunological Societies (IUIS) Junior Community was established to connect and empower early-career immunologists worldwide while showcasing their scientific contributions. Within this framework, the Research Topic “*IUIS Junior Community: Pushing the frontiers of immunology”* provided a dedicated platform for junior investigators to present innovative work spanning diverse areas of basic, translational, and clinical immunology. The aim of the Research Topic was to highlight work where early-career researchers have contributed as either a first or a senior author. We are pleased to present three original research papers and two review articles that highlight cutting-edge data and forward-looking perspectives from the next generation of immunologists.

Cruz-Cárdenas et al. report on the generation of clustered regularly interspaced short palindromic repeats (CRISPR)/Cas9-engineered neutrophil-like cell lines that lack either CD16b or CD32a, overcoming a major barrier to dissecting receptor-specific Fc gamma receptor (FcγR) signaling, cytokine regulation, and reactive oxygen species (ROS) generation in neutrophils. By establishing CD16b^−/−^ and CD32a^−/−^ promyelocytic lines that differentiate into neutrophil-like cells, they provide a donor-independent platform to interrogate the function of FcγRs, revealing distinct cytokine signatures and offering a powerful genetic system for mechanistic immunology and therapeutic Fc engineering (1).

Sanou et al. introduce the International ImMunoGeneTics Information System^®^ (IMGT)/monoclonal antibodies knowledge graph (IMGT/mAb-KG)—a structured, interoperable graph that integrates therapeutic mAb data with IMGT genomic and structural resources (2). This resource comprises over 100,000 triples and covers nearly 1,500 mAbs, hundreds of targets and indications, and approximately 40,000 study products. It enables sophisticated, cross-cutting queries on constructs, clinical development, mechanisms of action, and paratope–epitope relationships, thereby providing a robust informatics substrate for computational immunology and mAb discovery workflows.

Mentucci et al. present a comprehensive review of how cancer-associated fibroblasts (CAFs) impair dendritic cell (DC) biology across solid tumors, framing CAF–DC crosstalk as a key stromal axis of immune escape and a promising target for next-generation immunotherapy. By integrating DC subset biology with tumor microenvironment remodeling, they show how CAF-driven DC dysfunction skews the adaptive immunity toward tolerance and regulatory phenotypes (3). They further propose that effective restoration of DC function will require the combination of DC-targeted strategies and stromal/CAF reprogramming to improve the responses to vaccines, checkpoint blockade, and radiotherapy.

Ruffinatto et al. review the evidence positioning hematopoietic stem cells (HSCs) as a central reservoir of innate immune memory, emphasizing early hematopoiesis with myeloid bias as a key determinant of the long-term innate responses to infectious and inflammatory challenges (4). By synthesizing data on how cytokines, pathogen-associated molecular patterns (PAMPs), pathogens, and vaccines imprint HSCs, they link the specific inflammatory environment to durable changes in lineage bias, function, and HSC exhaustion. Thus, they propose that targeted modulation of “HSC training” could be exploited to enhance protective trained immunity or to mitigate maladaptive inflammatory memory in the context of chronic disease and transplantation (5).

Zhou et al. conducted an epidemiological study to further characterize varicella zoster virus infections in vaccinated individuals. They analyzed a 4-year epidemiological dataset collected in southwestern China to identify differences in infections with or without vaccination via regression modeling. They found that breakthrough infections were more likely to occur after receiving one vaccine dose and in the youngest age group. Their findings highlight the reduction of vaccine breakthrough cases in recipients of two vaccine doses, in line with the recently updated recommendations of the World Health Organization (6). They also point out the need to explore the potential benefits of further booster vaccinations. Mechanistic insights into such questions could benefit from serological and laboratory analyses in order to further improve our understanding on how vaccination shapes the disease transmission dynamics (7).

Taken together, this first IUIS Junior Community Research Topic showcases impactful advances by early-career immunologists in cellular and antibody engineering, immunoinformatics, tumor microenvironment, myeloid biology, stem cell-based innate immune memory, and epidemiology while acknowledging that many additional breakthroughs from junior investigators remain to be captured. Building on this momentum, submissions are now welcomed for “IUIS Junior Community: Research Topic—Volume II,” which aims to further amplify the scientific voice and visibility of the next generation of immunologists. While our IUIS Junior Community continues to grow, we hope for even more geographical and topical diversity for the next submissions and to continue highlighting the excellent work that is done by immunologists around the world.

## Author’s note

The scientific contributors to this Research Topic were community-based submissions based on their distinctive, pioneering contributions to immunology and their role as early-career corresponding or senior (last) authors. The work presented in these articles is intended to inform colleagues across the field and to enhance recognition of the innovative science being driven by the next generation of immunologists.
